# Network Plasticity and Intraoperative Mapping for Personalized Multimodal Management of Diffuse Low-Grade Gliomas

**DOI:** 10.3389/fsurg.2017.00003

**Published:** 2017-01-31

**Authors:** Cristina Diana Ghinda, Hugues Duffau

**Affiliations:** ^1^Department of Neurosurgery, The Ottawa Hospital, Ottawa Hospital Research Institute, Ottawa, ON, Canada; ^2^Neuroscience Division, University of Ottawa, Ottawa, ON, Canada; ^3^Department of Neurosurgery, Hôpital Gui de Chauliac, Montpellier University Medical Center, Montpellier, France; ^4^Brain Plasticity, Stem Cells and Glial Tumors Team, National Institute for Health and Medical Research (INSERM), Montpellier, France

**Keywords:** awake surgery, functional brain mapping, intraoperative mapping, anatomofunctional connectivity, low-grade gliomas, neuroplasticity, direct electrical stimulation

## Abstract

Gliomas are the most frequent primary brain tumors and include a variety of different histological tumor types and malignancy grades. Recent achievements in terms of molecular and imaging fields have created an unprecedented opportunity to perform a comprehensive interdisciplinary assessment of the glioma pathophysiology, with direct implications in terms of the medical and surgical treatment strategies available for patients. The current paradigm shift considers glioma management in a comprehensive perspective that takes into account the intricate connectivity of the cerebral networks. This allowed significant improvement in the outcome of patients with lesions previously considered inoperable. The current review summarizes the current theoretical framework integrating the adult human brain plasticity and functional reorganization within a dynamic individualized treatment strategy for patients affected by diffuse low-grade gliomas. The concept of neuro-oncology as a brain network surgery has major implications in terms of the clinical management and ensuing outcomes, as indexed by the increased survival and quality of life of patients managed using such an approach.

## Introduction

Neurosurgical resection remains the standard of care for gliomas, and the extent of resection (EOR) is one of the most important factors affecting the patients’ survival and quality of life for both high- and low-grade gliomas (1–9). The diffuse low-grade gliomas (DLGGs) portray a distinct clinical and radiological behavior and display particular gene expression signatures. DLGG is thought to represent a chronic invasive lesion that migrates along the white matter pathways, and eventually undergoes malignant transformation leading ultimately to death (10).

The concept of individualized surgery in neuro-oncological treatment of glial tumors is based on the goal of achieving a maximal tumor resection without inducing new neurological deficits. Analogously, for tumors located in proximity to critical functional areas, the use of intraoperative cortical and subcortical electro stimulation mapping (IEM) during awake craniotomy evolved over time and allows a substantial increase in the survival and quality of life of patients (1, 6, 9, 11–14).

The joint efforts of neuroscientists, researchers, and clinicians have provided an unprecedented ability to localize lesions and to assess the human brain function at the microscopic, mesoscopic, and macroscopic scales (15). The resultant array of invasive and non-invasive measures allowed surgeons to push the boundaries of safe surgical resection with subsequently improved clinical outcomes. As depicted by the extensive work performed by several research groups, the concepts of brain connectome and brain plasticity represent promising notions that advanced the neurosurgical treatments available for neurosurgical patients affected by DLGG.

### A Dynamic Concept: Tumor Growth and Functional Neuroplasticity

The survival benefit associated with an increased EOR has been demonstrated for both high- and low-grade gliomas; however, such oncological benefits need to take into account the risks of inducing neurological deficits. Although it is acknowledged that in DLGG, tumor infiltration follows the white matter tracts beyond the boundaries visualized on standard neuroimaging techniques, current treatment thresholds are still based on static radiological perceived boundaries. For instance, conventional radiotherapy protocols target a 2 cm conventional Euclidean distance around the macroscopically visible tumor, without taking into account the infiltrative and dynamic growth patterns of the lesion, thus equally radiating “non-cancerous brain tissue that could not only cause neurological deficits but also restrict the residual plasticity potential while leaving alive cancerous cells in other areas” (15).

One of glioma’s hallmark properties is the ability of cancer cells to invade healthy tissue, extensive attempts having been made both on the microscopic and macroscopic scales in order to determine the underlying pathophysiology. The different mechanisms involved in the plasticity of tumor cell motility have already been summarized by Taddei et al. and Cuddapah et al. (16, 17). Among the different structural and cellular adaptive strategies displayed by cancer cells, enhanced cell motility as well as resistance to hypoxia and acidity represent some of the key factors allowing tumors to elude antineoplastic drugs and radiotherapy treatments. From a biological perspective, the migration and invasion of tumor cells requires an increase in cellular motility, which involves formation of actin-based dynamic protrusions of the plasma membrane (18–20). Actin represents one of the key cytoskeletal filaments and its instability caused by hypoxia or tissue injury can facilitate entry of the cell into mitosis, thereby acting as an epigenetic determinant of the cell fate (21). Also, tumor cell motility can be modulated by acidity as the assembly of actin filaments in migrating cells increases with an intracellular pH higher than 7.2 (16). Moreover, *in vivo* imaging of membrane tube development over time revealed that the microtube-connected astrocytoma cells create a multicellular anatomical network that serve as routes for brain invasion, proliferation, and communication over long distances (18, 19). Disconnection of astrocytoma cells by targeting their tumor microtubes was already proposed as a possible new therapeutic strategy against cancer (22). Ion channels and transporters also appear to play a major role in the invasion strategies by mediating the hydrodynamic shape and volume changes displayed by tumor cells (17, 23–26). For instance, K+ and Cl− ions are thought to function as osmotically active ions that facilitate the dynamic cytoplasmic volume regulation occurring in tumor cells as they migrate and invade the surrounding tissue (25, 26).

The diffuse invasion exhibited by cancer cells can follow the same “extracellular routes of migration that are traveled by immature neurons and stem cells,” which similarly migrate along extracellular routes such as intracranial vasculature and white matter tracts (17, 27). Although the origin of gliomas is still unknown, it likely represents a complex phenomenon involving both genetic and epigenetic factors with a suspected cellular origin from a neural stem cell or an oligodendrocyte precursor cell (27–29). In addition, tumor recurrence occurs predominantly at the primary location of the tumor for both low- and high-grade gliomas. Tumor relapses might be linked to the presence of a cell subpopulation with stem cell characteristics, labeled as glioma stem cells (29, 30). While multiple studies assessed the presence of tumorigenic stem cells in high-grade lesions, the occurrence of those cells has equally been reported in patients harboring LGG (30). These cells are highly resistant to conventional chemotherapeutic drugs and could equally mediate tumor recurrence following radiation therapy (31–33). Tumor cell dissemination and heterogeneity represent important aspects that should be taken into account in order to improve the medical and surgical therapeutic regimens (34). Computational models attempt to simulate the functional consequences associated with brain tumor growth by incorporating the tumor-induced plastic compensatory mechanism along with the structural and biological heterogeneity of gliomas (35).

**Figure 1:**
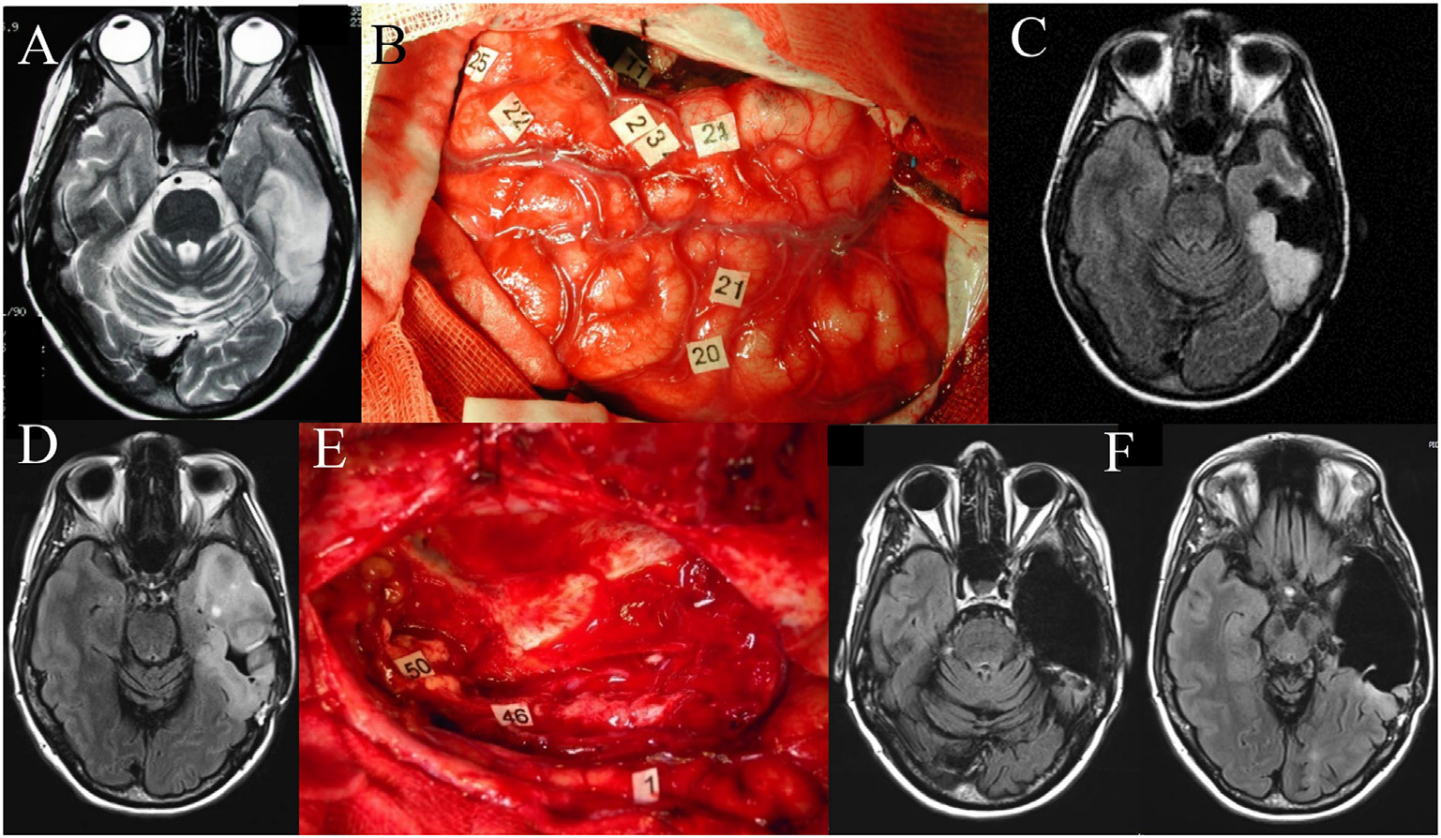
**Left temporal diffuse low-grade glioma (DLGG)**. Axial FLAIR-weighted MRI **(A)** showing a left temporal DLGG in a 36-year-old patient who presented with isolated seizures and no neurological deficits. Intraoperative photograph during the first awake surgery **(B)**, after resection was performed according to individual functional boundaries. Stimulation mapping demonstrated the persistence of eloquent cortical areas in the temporal lobe (tags 22, 23, 24, 25) as well as subcortical fibers (tag 11, corresponding to the inferior longitudinal fascicle) still critical for language function. Postoperative axial FLAIR-weighted MRI **(C)** revealing a partial resection, with a posterior residual tumor voluntarily left for functional reasons. The diagnosis of DLGG was confirmed, and the patient resumed a normal familial, social, and professional life. Ten years later, epileptic seizures recurred concomitantly with an imaging progression as demonstrated on the axial FLAIR-weighted MRI **(D)**. Reoperation was proposed to the patient. Intraoperative photograph **(E)** during the second awake surgery, after resection was performed according to the new individual functional boundaries. Electrocortical stimulation mapping revealed brain reorganization, allowing the achievement of a significantly wider resection compared to the first surgery. Of note, at the subcortical level, stimulation of the left inferior fronto-occipital fascicle (IFOF) (46 and 50) elicited semantic paraphasia when stimulated at the end of surgery. Thus, resection of the anterior part of the inferior longitudinal fascicle was possible given the compensation provided by the direct ventral pathway represented by the left IFOF. Postoperative axial FLAIR-weighted MRI **(F)** performed 3 months after the second surgery showing a complete resection, made possible due to mechanisms of neuroplasticity, in a patient who returned to a normal life with no permanent neurological deficit.

Delineating the extent of tumor infiltration has been subject to intense research, as the boundaries between tumor and healthy tissue are difficult to detect macroscopically with current imaging techniques like functional MRI (fMRI), positron emission tomography, spectroscopy, and diffusion tensor imaging (36–38). In the case of tumor-related epilepsy, such techniques allowed to establish a link between the peritumoral tissue and the tumor-related epileptogenesis, which can explain both the antiepileptic effects of oncological treatments (39–41) and the increase in seizure frequency as tumors progress (42). As both infiltrated peritumoral tissue and connectivity changes have been related to the development of seizures, understanding brain reorganization mechanisms has important clinical implications for controlling refractory seizures (43, 44). Recent studies investigated the role of functional network synchronizability to predict spread of seizures before they begin and also described control regions that strongly synchronize or desynchronize network dynamics (45). By investigating time-varying functional networks, the dynamic changes in the topographical organization of different functional networks could have wide applicability in mapping the plastic reorganization occurring in other diseases such as stroke and trauma (46–51). Similarly, brain tumors may also induce changes in large-scale functional connectivity (FC) that should be taken into account by the surgical approach (52). For instance, the complex language network reorganization occurring in the setting of a dominant left hemisphere DLGG infiltrating classical “Broca” and “Wernicke” areas (53–55) allow tumor resection with no functional consequences as depicted in the case illustrated in Figure 1. Thus, understanding the underlying neuromodulation principles governing the neurosynaptic networks could lead to new methods for functional restoration (48, 49, 53).

Cerebral plasticity represents the “continuous process allowing short-term, middle-term, and long-term remodeling of neurosynaptic maps, to optimize the functioning of brain networks” (56). The concept of adult neuroplasticity exemplifies the strong interplay between the cortex and other structures provided by the myriad of cortical and subcortical connections. The underlying mechanisms for this functional reorganization and brain plasticity are not fully elucidated, and multiple theories have been proposed such as modulation of synaptic efficacy, neurogenesis, cortical hyperexcitability, redistribution, unmasking of latent networks, and establishment of new functional connections (51, 57–61). Although mounting evidence depict functional reorganization in the setting of a surgical intervention, the concept of brain plasticity in the context of DLGG is still controversial (62). Nonetheless, our current understanding of the morphological, biochemical, and connectivity changes occurring in the setting of a tumor is still in its infancy and long-term large multicenter studies incorporating longitudinal multimodal investigations will likely allow us to gather more objective evidence and improve our understanding of the underlying mechanisms.

This approach facilitates the concepts of “functional neuro-oncology” and of “preventive glioma surgery” in order to achieve earlier and more complete resections, while giving the patients the opportunity to enjoy a normal life. Understanding the individual organization of the cortical and subcortical connectivity is essential to optimize the risk–benefit ratio of glioma surgery (63). Prominent experts in this field suggest an integration of the conceptual achievements in the neuroscience, neuroimaging, and genetic fields, in order to create a holistic personalized treatment strategy incorporating “the course of this chronic disease, reaction brain remapping, and oncofunctional modulation elicited by serial treatments” (10).

### Connectomics and Glioma Surgery

Functional connectivity is a measure used to express the degree of communication between brain areas and thus to describe brain networks (64). One of the main proposed mechanisms of adult plasticity reposes on the connectome concept where the brain processing relies on “dynamic large-scale, parallel subcircuits able to interact and to compensate themselves following cerebral injury” (65, 66). The concept of brain connectome depicts the dynamical structural and functional neural networks that form at multiple spatial and temporal scales (67). While it is possible to portray structural networks delineating anatomical connectivity with deterministic tractography-derived fiber tracts (68), “functional networks” are derived from statistical estimates of time series data such as resting-state fMRI (69). For instance, using multimodal magnetic resonance images derived from the Human Connectome Project, Glasser and colleagues performed a multimodal parcelation of distinct cortical areas using an objective, semiautomated neuroanatomical approach and a robust machine-learning classifier (70). Although such non-invasive imaging studies outline a detailed non-invasive mapping of the macroscopic functional connectome, it provides just one view of the “complete” brain connectome and cannot provide the direct neuronal activity flow available through electrophysiological techniques (67, 71).

Brain tumors alter the normal structural and FC of the brain, consequently impacting the normal functioning of the brain. Altered FC in patients with brain tumors affects not only the tumor area but also other brain areas, as demonstrated through different imaging modalities (72–77). For instance, changes in resting-state networks in patients with tumors localized in the left hemisphere were observed in the contralateral side and correlated with alterations in some cognitive functions even before the onset of major symptoms (74). Intrinsic FC measures can also predict surgical outcome, and thereby could “provide information regarding the residual presence of function and also could define the extent of brain tumor invasion that may not be evident on structural MRI” (78).

As described by De Benedictis and Duffau, the classical “topological” representation ought to be replaced by a “hodotopical” framework, which takes into account the changes occurring in the large-scale networks of the brain (65). Only by acknowledging the complex cortico-subcortical network of the brain, the clinicians could further understand and take into account the dynamical neural processes occurring at distinct spatial and temporal scales (79). The functional connectome framework refined our understanding of functional localization as evidenced through the contemporary concepts of language organization, namely, that neuronal groups participate as components of a network allowing reorganization and recruitment of parallel circuits in the setting of injury (80–83).

Considering glioma surgery as “brain networks surgery” has led not only to a dramatic decrease of permanent neurologic impairment (<2% in series using intraoperative cortico-subcortical mapping) but also to improvement of higher order functions such as working memory, neurocognitive functions, and emotions and behavior, as evidenced by postoperative neuropsychological assessments following surgery (84). Therefore, the concept of neuro-oncology as a brain network surgery has major implications in terms of the clinical management and ensuing outcomes, as indexed by the increased survival and quality of life of patients managed using such an approach.

### Awake Craniotomy and Intraoperative Mapping

Although the art and science of brain mapping was once the purview of epilepsy surgeons, the use of this technique in the neuro-oncological field had seen an exponential increase over the last decades. Proper choice and execution of brain mapping techniques has improved the precision and safety of the surgical treatment for some of the most challenging cases and can currently allow a more radical surgical resection than indicated by presumed preoperative functional localization. This entails an optimization of the intraoperative tests’ selection based on the functional cortico-subcortical networks expected to be encountered as well as on the specific preoperative neurological and neuropsychological assessments of each patient (85–87).

Although many promising brain mapping techniques are currently being refined, the large interindividual differences between healthy and diseased brain preclude the ability to reliably identify standard imaging-based biomarkers for functional connectomics (88, 89). As such, cortical and subcortical mapping via direct electrical stimulation continues to remain the most reliable approach for accurate localization of highly functional centers specific to each patient; the usefulness of this technique being described even for children (90). Furthermore, the continuous assessment of cognitive and neurophysiological parameters provides the neurosurgeon with immediate feedback on the impact of his/her intervention.

The concept of “eloquent” cortex depends on the view that, although all cortical areas are “capable of being engaged in useful function, some brain regions are clearly more critical than others” (91), causing some degree of functional decline if resected or disconnected. This framework shift has direct implications in the clinical practice as the presumed eloquence represents a modifiable risk factor for survival (92, 93). Although a detailed knowledge of both cortical and subcortical anatomical structures represents the cornerstone of neurosurgery, understanding the underlying functional correlations provide the fine details of the relationship between the lesion to be managed and the healthy brain (94). Figure 1 shows an illustrative case depicting the importance of performing intraoperative mapping of cortical and subcortical fibers in a patient with a left temporal DLGG. As portrayed, the respect of functional boundaries during the first surgery allowed the patient to enjoy a normal life for 10 years, whereas the language network reorganization occurring in the setting of a slowly growing tumor allowed subsequent resection of tumoral tissue at sites where functionality prevented tumor resection initially (Figure 1).

Despite the fact that the precise influence on the electrophysiological state of brain’s networks and the biophysical modeling of the electrode–tissue interface is not well elucidated (95), direct electrical stimulation represents a highly reliable and reproducible technique (58, 95–101). IEM has equally allowed to increase the quality of life for patients affected by a glioma in the non-dominant hemisphere by testing functions such as spatial awareness (102) or even mentalizing (103) to avoid injuring the networks involved in those functions. Furthermore, IEM during awake craniotomy allows the unique opportunity to assess and validate the anatomo-functional connectivity for multimodal systems such as sensorimotor, language, visuospatial, and sociocognitive systems (82) providing a real-time understanding of the individual organization of both cortical epicenters and subcortical connectivity (104). We can envisage the future development of platforms allowing neurosurgeons to link the intraoperative cortical stimulation results with macroscopic neuronal network models and use connectivity-based modeling to predict functional changes.

Several manuscripts provide a comprehensive overview of the methods and technical nuances proposed for a maximal safe resection during awake brain tumor surgery (105–107). There is increasing evidence that this technique allows to improve the outcomes by maximizing the EOR while preserving functional cortex in both low- and high-grade gliomas (1, 6, 9, 108, 109). Other benefits associated with this technique are shorter hospital stay, less blood loss, shorter operative time, reduced pain and anxiety, cost effectiveness, as well as lower complications and morbidity (110, 111). Nonetheless, careful preoperative planning by a dedicated multidisciplinary team with an informed patient remains an important prerequisite for a successful awake craniotomy (90).

## Conclusion

The paradigm shift encouraging the translation of the most recent findings in the field of neurological science to the clinical setting allowed a better understanding of the interactions between the infiltrative and dynamic growth patterns of DLGG and brain adaptation mechanisms (such as neuroplasticity and network reorganization). Using multimodal imaging studies and different neurophysiological tools does not take the place of a meticulous surgical technique and an extensive knowledge of the functional–structural anatomy in order to protect the cortical and subcortical FC. The concept of awake craniotomy as a brain network surgery allows neurosurgeons to adequately assess the dichotomy between the neurological and oncological risk management. It also highlights the delicate function–oncological balance that needs to be maintained, as it will ultimately reflect on the quality of life and overall survival rate of the patients. A joint multidisciplinary approach where the emerging advancements from different fields are integrated in clinical practice in a personalized dynamic approach using ongoing feedback from clinical–radiological monitoring could provide more effective treatment options for patients affected by DLGG as already demonstrated by the increased survival and quality of life of patients treated using such a treatment strategy.

## Author Contributions

All authors have made substantial, direct, and intellectual contribution to the work and approved it for publication.

## Conflict of Interest Statement

The authors declare that the research was conducted in the absence of any commercial or financial relationships that could be construed as a potential conflict of interest.
